# Regulation of voltage-gated potassium channels attenuates resistance of side-population cells to gefitinib in the human lung cancer cell line NCI-H460

**DOI:** 10.1186/s40360-017-0118-9

**Published:** 2017-02-21

**Authors:** Seon Young Choi, Hang-Rae Kim, Pan Dong Ryu, So Yeong Lee

**Affiliations:** 1 Laboratory of Veterinary Pharmacology, College of Veterinary Medicine and Research Institute for Veterinary Science, Seoul National University, Seoul, Korea; 2 Department of Anatomy and Cell Biology, and Biomedical Sciences, College of Medicine, Seoul National University, Seoul, Korea

**Keywords:** Lung cancer cell, Drug resistance, Side population, Voltage-gated potassium channel, Combination therapy

## Abstract

**Background:**

Side-population (SP) cells that exclude anti-cancer drugs have been found in various tumor cell lines. Moreover, SP cells have a higher proliferative potential and drug resistance than main population cells (Non-SP cells). Also, several ion channels are responsible for the drug resistance and proliferation of SP cells in cancer.

**Methods:**

To confirm the expression and function of voltage-gated potassium (Kv) channels of SP cells, these cells, as well as highly expressed ATP-binding cassette (ABC) transporters and stemness genes, were isolated from a gefitinib-resistant human lung adenocarcinoma cell line (NCI-H460), using Hoechst 33342 efflux.

**Results:**

In the present study, we found that mRNA expression of Kv channels in SP cells was different compared to Non-SP cells, and the resistance of SP cells to gefitinib was weakened with a combination treatment of gefitinib and Kv channel blockers or a Kv7 opener, compared to single-treatment gefitinib, through inhibition of the Ras-Raf signaling pathway.

**Conclusions:**

The findings indicate that Kv channels in SP cells could be new targets for reducing the resistance to gefitinib.

**Electronic supplementary material:**

The online version of this article (doi:10.1186/s40360-017-0118-9) contains supplementary material, which is available to authorized users.

## Background

Epidermal growth factor receptor (EGFR) is an oncogene that is involved in the development and progression of several human cancers, including non-small cell lung cancer (NSCLC). Approximately 10–30% of non-small cell lung cancer (NSCLC) patients have EGFR gene mutations. EGFR tyrosine kinase inhibitor (TKI) therapies are effective for NSCLC patients who have EGFR kinase domain mutations that target four exons (exon 18-exon 21) [1, 2]. Gefitinib (ZD 1839, Iressa), a small-molecule EGFR TKI, was approved by United States’ Food and Drug Administration (FDA) in 2003 for NSCLC, which comprises 80% of lung cancers [3]. Gefitinib sensitivity occurs in patients who have EGFR mutations, such as L858R or exon 19 deletion. However, a T790M-like secondary mutation in exon 19 of EGFR was also associated with resistance to gefitinib in NSCLC cells that contain the L858R-EGFR mutation [4]. Moreover, *KRAS* mutations also demonstrate resistance to gefitinib in vitro and in vivo [4, 5].

Accumulating evidence indicates that cancer stem cells (CSCs) have self-renewal properties in various solid tumors, and play a role in tumor development and progression [6, 7]. Side-population (SP) cells, a fraction of cancer stem cells, can be identified by efflux of Hoechst 33342 dye [8]. Moreover, SP cells have higher clonogenic potential and expression levels of ATP-binding cassette (ABC) transporters than main-population cells (known as Non-SP cells) [9, 10]. Several groups have suggested that SP cells that were dye-excluding cell portion in a tumor including lung cancer were responsible for anti-cancer drug resistance [11, 12]. SP cells in tumors possess phenotypes and signaling pathways similar to those of normal stem cells, which have high efflux of drugs [13, 14]. High expression levels of ABC transporters, especially ABCG2, in normal stem cells and tumor stem cells are considered to be responsible for drug resistance [15–17]. In various types of tumor, SP cells related to drug resistance have been isolated [18–20].

Recently, several reports have proposed that ion channels regulate the survival and growth of cancer stem cells [21, 22]. Silencing of chloride intracellular channel 1 (CLIC1), which is significantly overexpressed in stem/progenitor cells from human glioblastomas, reduced the proliferative and clonogenic capacity of stem/progenitor cells [21]. The transient receptor potential cation channel, subfamily M, member 7 (TRPM7) also leads to increased cancer stem cell proliferation in glioblastoma multiforme (GBM) through activation of the JAK2/STAT3 and/or Notch signaling pathways [22]. Moreover, blockade of CLIC1 induces apoptosis of 1,3-Bis(2-chloroethyl)-1-nitrosourea (BCNU)-resistant cancer stem cells of GBM [23].

However, research regarding the voltage-gated potassium (Kv) channel expression patterns of SP cells, and the involvement of Kv channels in reducing the resistance of SP cells to gefitinib, has not been reported.

Therefore, the present study was performed to compare Kv channel expression between SP cells and Non-SP cells in a gefitinib-resistant NCI-H460 cell line, which had wild-type EGFR and *KRAS* mutations [4], and to examine the inhibitory effect of combination treatment with gefitinib and Kv channel blockers or a Kv7 opener on the viability of gefitinib-resistant SP cells.

## Methods

### Cells and reagents

The human lung adenocarcinoma cell line (NCI-H460) was obtained from Korea Cell Line Bank (Seoul, Korea). The cells were maintained in complete growth medium supplemented with 10% fetal bovine serum (WelGene, Korea) and 1% antibiotics (Sigma-Aldrich, St. Louis, MO, USA) in an incubation system at 37 °C with 5% CO_2._ The cells were harvested using 1% trypsin-EDTA (Sigma-Aldrich) when they were in the logarithmic phase of growth, for SP analysis. Hoechst 33342 and fumitremorgin C (ABCG2 blocker) were purchased from Sigma-Aldrich. The anti-cancer drug gefitinib (Santa Cruz Biotechnology, CA, USA), tetraethylammonium (TEA, Sigma-Aldrich), 4-aminopyridine (4-AP, Sigma-Aldrich), and flupirtine (Tocris Bioscience, Bristol, UK) were used to blockade cell growth.

### Isolation of side population

The protocol was based on that of Goodell et al. [24]. Briefly, the NCI-H460 cells were re-suspended at 1 × 10^6^ cells/mL in pre-warmed RPMI 1640 (WelGene) with 2% fetal bovine serum. Hoechst 33342 dye was added at a final concentration of 5 μg/mL in the presence or absence of fumitremorgin C (10 μg/mL), and the cells were incubated in a 37 °C water bath for 90 min with intermittent shaking. At the end of the incubation, the cells were washed with ice-cold Hank’s Balanced Salt Solution (HBSS) (Sigma-Aldrich), centrifuged down at 4 °C, and resuspended in ice-cold HBSS. Propidium iodide (Sigma-Aldrich) at a final concentration of 2 μg/mL was added to the cells to gate the viable cells. The cell preparations were filtered through a 40-μm cell strainer (BD Biosciences, San Jose, CA, USA) to obtain a single cell suspension. Cells were analyzed and sorted into SP and Non-SP using BD FACSAriaIII® (BD Biosciences).

### Real-time RT-PCR analysis

Total RNA was extracted using a Hybrid-R prep kit (GeneAll, Korea), according to the manufacturer’s protocol. Reverse transcription was performed using M-MLV reverse transcriptase (Thermo Fisher Scientific, Fremont, CA, USA) and random primer (Promega, Madison, WI, USA) according to the manufacturer’s instructions. Real-time reverse transcription-PCR (real-time RT-PCR) was done with SYBR Green reagents (TAKARA, Japan) on Step-One Plus (Applied Biosystems, Foster City, CA, USA). Primers were designed to generate a PCR product. The relative mRNA expression level of the genes was normalized to GAPDH, and expressed as fold change relative to Non-SP cells. Table 1 represents the list of primers used for real-time RT-PCR.

**Table 1:** Primers for real-time RT-PCR

Gene (Accession number)	Sequences	Product size (base pair)
ABCG2 (NM_001257386)	F 5′-AGATGGGTTTCCAAGCGTTC-3′ R 5′-TGGTTGGTCGTCAGGAAGAA-3′	191
ABCC1 (NM_004996.3)	F 5′- ACTGCCTTGGGATTTTTGCT-3′ R 5′- CATGGTGATGCCCAAGAGAG-3′	135
OCT4 (NM_001173531)	F 5′- ATTTTGAGGCTGCTGGGTCT-3′ R 5′- CCTCAGTTTGAATGCATGGG-3′	205
NANOG (NM_024865)	F 5′-CAGAAAAACAACTGGCCGAA-3′ R 5′-GGTCTGGTTGCTCCACATTG-3′	147
Kv1.3 (NM_002232)	F 5′- TCTCCTTCGAACTGCTGGTG-3′ R 5′- ATGGCCACAATGTCGATCAG-3′	95
Kv1.4 (NM_002233.3)	F 5′-ACGAGGGCTTTGTGAGAGAA -3′ R 5′-TAAGATGACCAGGACGGACA- 3′	144
Kv4.1 (NM_004979)	F 5′-CTCCCTCAGCTCCTTCTTGG-3′ R 5′-GGGCAATGTTCTGAGGGACT-3′	97
Kv7.3 (NM_001204824.1)	F 5′-GGTGCAGGTCACGGAGTATT-3′ R 5′-GGGCTGACTTTGTCAATGGT-3′	174
Kv7.5 (NM_001160134.1)	F 5′-CGCTTTCGTTTTTCTCCTTG-3′ R 5′-CGAGCAAACCTCAGTCTTCC-3′	207
Kv9.3 (NM_002252.3)	F 5′-CAGTGAGGATGCACCAGAGA-3′ R 5′-TTGCTGTGCAATTCTCCAAG-3′	200
Kv10.1 (NM_002238.3)	F 5′-TGACCCCAAACTTATCCGCA-3′ R 5′-CTGCTGATGCCCTCATCCAC-3′	116
Kv11.1 (NM_001204798)	F 5′-GACGTGCTGCCTGAGTACAA-3′ R 5′-AGCCGAGTAGGGTGTGAAGA-3′	121
GAPDH (NM_002046.4)	F 5′-CTCTGCTCCTCCTGTTCGAC-3′ R 5′-ACGACCAAATCCGTTGACTC-3′	112

### Cell proliferation assay

A cell proliferation assay was performed using the Cell Counting Kit-8 (Dojindo, Japan). Sorted SP cells were plated in 96-well culture plates (SPL, Korea) at 5 × 10^2^ cells per well, and cultured in the complete growth medium. CCK-8 solution was added to each well at 0, 12, 24, 48, and 72 h. After 4 h of incubation, the absorbance was determined through a 450 nm filter in a microplate reader (TECAN, Männedorf, Switzerland), and the growth curve was plotted using optical density (OD) values. Each experiment was performed in triplicate.

### Inhibitory effect of gefitinib, TEA, 4-AP, and flupirtine on NCI-H460 cell viability

The cells were seeded in 96-well plates at a concentration of 1 × 10^3^ cells per well. After treatment with gefitinib, Kv channel blockers (TEA, 4-AP), and the Kv7 channel opener (flupirtine), the cell viability was measured using the 3-(4,5-dimethylthiazol-2-yl)-2,5-diphenyltetrazolium bromide (MTT) assay. Briefly, 0.5 mg/mL of thiazolyl blue tetrazolium (Sigma-Aldrich) was added to the cells, and the cells were then incubated for 4 h. The MTT formazan was dissolved with dimethyl sulfoxide (DMSO) (Sigma-Aldrich), and the absorbance at 570 nm was determined using a microplate reader. The results were presented as a percentage of the control values.

### Western blotting assay

The western blotting assay was applied to study the proteins related to the EGFR-Ras-Raf signaling pathway. Proteins were extracted and subjected to 10% SDS-PAGE, then transferred to PVDF membranes. The membranes were blocked with 5% skim milk in Tris-buffered saline with Tween™20 (Sigma-Aldrich) for 1 h at room temperature. The membranes were reacted with primary antibodies overnight at 4 °C. The primary antibodies were as follows: phosphorylated EGFR (1:500); Ras (1:500); phosphorylated Raf (1:500); phosphorylated Erk1/2 (1:500, all from Cell Signaling Technology, Danvers, MA, USA); and β-actin (Santa Cruz Biotechnology, 1:1,000). β-actin protein was used as the control.

### Statistical analysis

Data were generally presented as the mean ± SE, and the statistical differences between experimental groups were analyzed with Student’s *t*-test using the statistical software Origin 8.0. A *p* value of < 0.05 was considered statistically significant in all cases.

## Results

### Inhibition of viability of NCI-H460 cells by blockade and opening of Kv channels

**Fig. 1:**
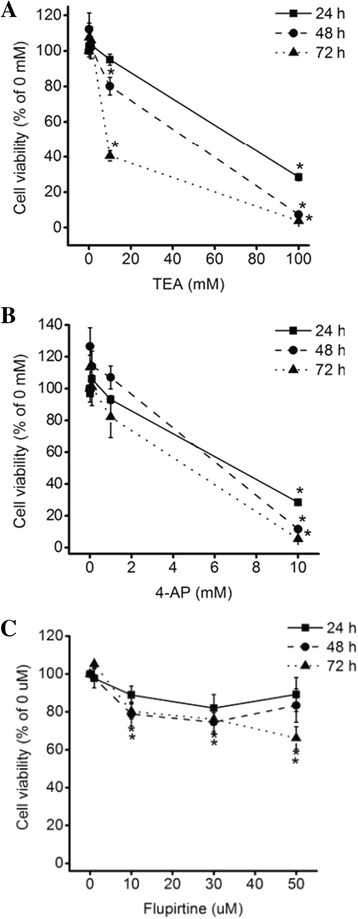
Effect of Kv blockers and an opener on NCI-H460 cell viability. Cell viability of NCI-H460 cells treated with **a** TEA **b** 4-AP, or **c** flupirtine for 24, 48, and 72 h was decreased in a dose-dependent manner. Results are mean ± SE of triplicate experiments, (*) *p* < 0.05

In order to determine the inhibitory effect of Kv channel blockers (TEA and 4-AP) and Kv7 opener (flupirtine) on the viability of NCI-H460 cells, cell viability was determined using an MTT assay. Cells treated with TEA, 4-AP, and flupirtine at different concentrations were incubated for 24, 48, and 72 h. The results indicated that the viability of NCI-H460 cells treated with TEA, 4-AP, and flupirtine was decreased in a dose-dependent manner (Fig. 1).

### Isolation of SP cells within the NCI-H460 cell

**Fig. 2:**
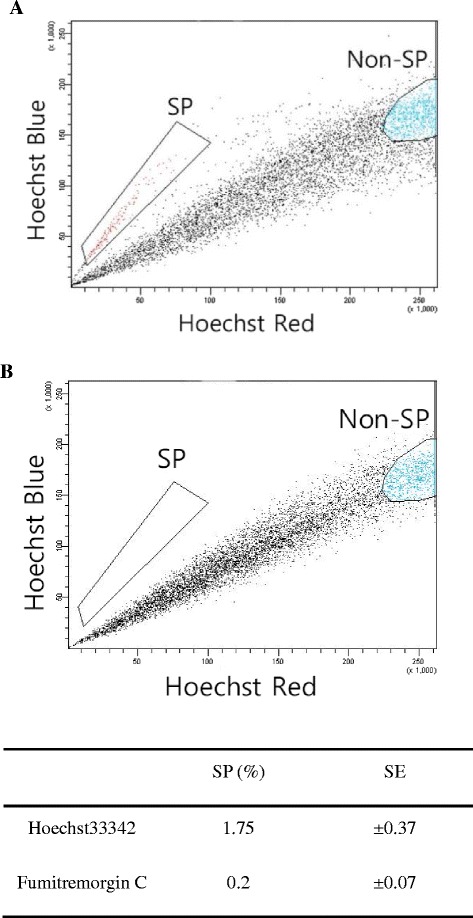
Isolation of SP cells in a cultured NCI-H460 lung cancer cell line. SP cells plotted in the absence (**a**) or presence (**b**) of fumitremorgin C. Results are mean ± SE of triplicate experiments, (*) *p* < 0.05

In order to isolate the SP cells, the NCI-H460 cells were stained with Hoechst 33342, which was extruded by ABC transporters in stem-like cells. Next, we quantified the SP cells with dual-wavelength flow cytometry. The results show that SP cells were detected at 1.75% in multiple independent experiments (*n* = 34), as illustrated in Fig. 2a. SP cells were also eliminated in the presence of fumitremorgin C, which is specific blocker of ABCG2 (Fig. 2b). According to our results, SP cells, which had dual negative wavelengths of blue and red, were isolated in gefitinib-resistant NCI-H460 cells.

### Characterization of SP cells

**Fig. 3:**
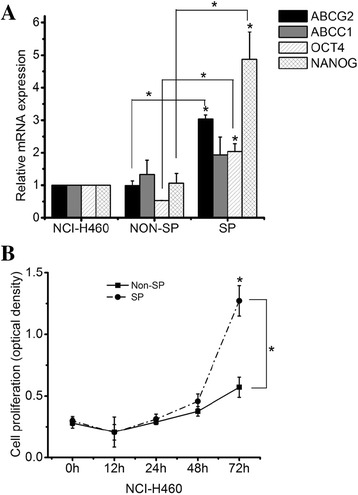
Characterization of SP cells in NCI-H460 lung cancer cells. **a** Real-time RT-PCR analysis of cancer stem cell marker genes **b** showing proliferation of sorted SP cells and Non-SP cells. Results are mean ± SE of triplicate experiments, (*) *p* < 0.05

In order to confirm the characteristics of the SP cells, the mRNA expression levels of marker genes were identified using real-time RT-PCR, and compared to Non-SP cells. The mRNA expression level of ABCG2 was significantly increased in the SP cells. The mRNA expression levels of OCT4 and NANOG, which are specific markers of self-renewal, were significantly elevated in the SP cells (Fig. 3a). Also, to determine the differences in proliferation potential, the proliferation of SP and Non-SP cells was measured using the CCK-8 assay. After the sorted SP and Non-SP cells were incubated overnight, the CCK-8 assay was performed at 0, 12, 24, 48, and 72 h. The result reveals that the proliferation potential of SP cells was also significantly 2 times higher than Non-SP cells at the 72-h time-point (Fig. 3b).

### Comparison of mRNA expression level of Kv channels between SP- and Non-SP cells

**Fig. 4:**
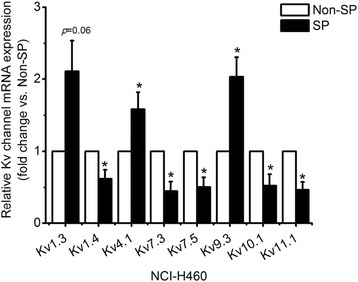
Comparison of Kv channel expression between sorted SP cells and Non-SP cells. Real-time RT-PCR analysis of Kv channel subtypes. Results are mean ± SE of triplicate experiments, (*) *p* < 0.05

To determine the mRNA expression level of Kv channel subtypes in SP cells, mRNA expression levels were measured with a real-time RT-PCR. The result shows that the mRNA expression levels of Kv channel subtypes in SP cells were significantly different compared to Non-SP cells. The expression levels of Kv1.4, Kv7.3, Kv7.5, Kv10.1, and Kv11.1 mRNA in SP cells were less than in Non-SP cells. In other types of Kv channels, mRNA expression levels of Kv4.1 and Kv9.3 in SP cells were 1.58- and 2.03-fold higher, respectively, than in Non-SP cells (Fig. 4). The mRNA expression level of Kv1.3 between the SP and Non-SP cells was not significantly different (*p* = 0.06). This result indicates that the expression patterns of Kv channel subtypes are different between SP cells and Non-SP cells.

### Reduction of resistance to gefitinib with Kv channel blockers and opener in SP cells

**Fig. 5:**
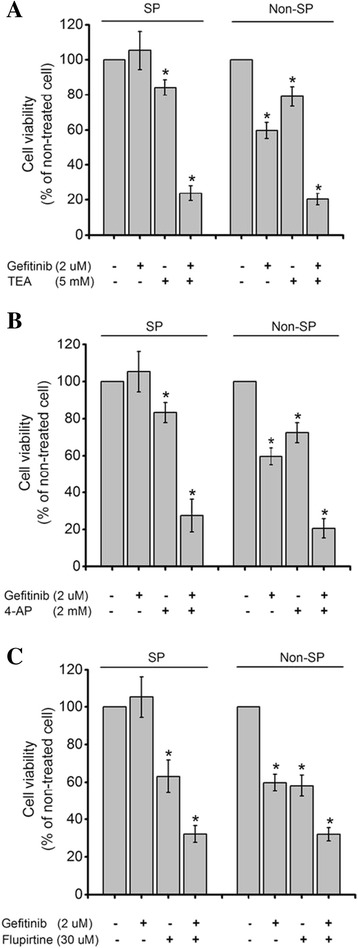
Inhibitory effect of combination treatment with gefitinib and Kv blockers (TEA and 4-AP) or an opener (flupirtine) on sorted SP cells and Non-SP cell viability. Cell viability of SP cells and Non-SP cells treated with **a** TEA 5 mM **b** 4-AP 2 mM, or **c** flupirtine 30 μM together gefitinib 2 μM. Results are mean ± SE of triplicate experiments, (*) *p* < 0.05

To evaluate their inhibitory effects of gefitinib, Kv channel blockers and Kv7 opener on viability of the sorted SP cells, the viability of sorted cells treated with Kv channel blockers, Kv7 opener, and gefitinib for 72 h was estimated. The results showed that the viability of SP cells treated with gefitinib alone at an insensitive concentration (2 μM) [4] was not decreased. Compared to the SP cells, the viability of Non-SP cells treated with gefitinib alone at a concentration of 2 μM was decreased by 40.3% (Fig. 5). In cells treated with gefitinib at a concentration of 1 μM, viability was not decreased in the SP and Non-SP cells. An additional file shows this in detail (see Additional file 1).

In addition, the viability of SP cells treated with TEA alone at a concentration of 5 mM or with a combination treatment of TEA and gefitinib at concentrations of 5 mM and 2 μM, respectively, was decreased by 15.8 and 76.16 (Fig. 5a). After treatment with 4-AP alone at a concentration of 2 mM, or a combination treatment of 4-AP and gefitinib at concentrations of 2 mM and 2 μM, the viability of the SP cells was decreased by 16.7 and 72.6%, respectively (Fig. 5b). The viability of the SP cells treated with flupirtine alone at a concentration of 30 μM, or with a combination treatment of flupirtine and gefitinib at concentrations of 30 μM and 2 μM, was decreased 37 and 69.4%, respectively (Fig. 5c). According to the results, SP cells were less sensitive to gefitinib than Non-SP cells, and the viability of SP cells with combination treatments was decreased more than with gefitinib as a single treatment. However, the degree of reduction between SP and Non-SP cells in each group was not different. Table 2 summarizes the percentage of decreased viability of SP and Non-SP cells from combination and single treatments.

**Table 2:** Viability of SP- and Non-SP cells treated with gefitinib and Kv channels blocker or Kv7 opener

Cell viability (%)	Gefitinib (2 μM)	TEA (5 mM)	4-AP (2 mM)	Flupirtine (30 μM)	TEA (5 mM)	4-AP (2 mM)	Flupirtine (30 μM)
					(Gef 2 μM)		
Non-SP	59.68 (±4.54)	79.09 (±5.62)	72.46 (±5.45)	58.08 (±5.67)	20.39 (±3.27)	20.54 (±5.22)	32.02 (±3.48)
SP	105.32 (±10.92)	84.22 (±4.40)	83.32 (±5.43)	63.00 (±8.81)	23.84 (±4.27)	27.44 (±8.87)	30.63 (±3.95)

### Inhibition of activated EGFR-Ras-Raf-Erk signaling pathway with combination treatment

**Fig. 6:**
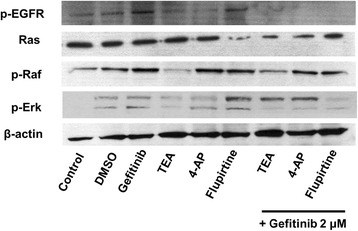
Inhibition of the EGFR-Ras-Raf signaling pathway on NCI-H460 cells with combination treatment. The protein expression level of p-EGFR, total Ras, p-Raf, and p-ERK1/2 was inhibited in cells with gefitinib and Kv blockers (TEA and 4-AP) or an opener (flupirtine)

To determine the signaling pathway involved in gefitinib resistance in cells with the combination treatment, western blotting to detect proteins of the EGFR-Ras-Raf signaling pathway was performed. The result showed that phosphorylated EGFR (p-EGFR) protein in cells with the combination treatment was decreased, compared to single-treatment gefitinib or blockers (Fig. 6). Also, total Ras protein in cells with the combination treatment was decreased, compared to single treatment with a gefitinib or blockers. Phosphorylated Erk (p-Erk) protein in cells with the combination treatment was decreased as compared to single treatments with 4-AP and flupirtine. However, the expression level of phosphorylated Raf (p-Raf) protein was not different between the combination treatment and the single treatment. According to the results, combination treatment with gefitinib and Kv channel blockers or the Kv7 opener reduced the viability of gefitinib-resistant NCI-H460 cells through inhibition of the EGFR-Ras-Raf-ERK pathway.

## Discussion

In the present study, isolated SP cells responsible for resistance to gefitinib in the NCI-H460 cell line demonstrated different Kv channel expression patterns compared to Non-SP cells. Our results also indicated that the viability of SP cells treated with gefitinib and Kv channel blockers or the Kv7 opener was more significantly inhibited than with single treatments through inhibition of the activated EGFR-Ras-Raf-Erk signaling pathway.

Several research groups have suggested that SP cells (known as cancer stem cells) were isolated from various cancer cell lines, including lung cancer, at various frequencies [12, 25–27]. The frequency of isolated cancer stem cells from human cancers was infrequent [28]. In our study, SP cells in gefitinib-resistant lung cancer cells were isolated (1.87%). Moreover, SP cells had characteristics similar to those of normal stem cells that were responsible for drug resistance [13, 15]. SP cells in tumor had high expression of ABC transporters and a high proliferative capacity, similar to normal stem cells [29, 30]. Some reports suggest that ABCG2 regulates self-renewal and stem cell marker expression in radiation-resistant glioma cells and NSCLC cell lines [31, 32]. Furthermore, other studies have proposed that gefitinib is a substrate extruded by ABCG2 and that a high expression of ABCG2 is responsible for acquired resistance to gefitinib [33–35]. Corresponding to previous studies, we isolated SP cells with high mRNA expression of ABCG2, OCT4, and NANOG genes and high proliferative potential from gefitinib-resistant NCI-H460 lung cancer cells. Moreover, SP cells in the NCI-H460 cell line demonstrate less sensitivity to gefitinib than Non-SP cells.

Potassium channels are involved in the regulation of anti-cancer-drug resistance. The up regulation of Kv1.5 increased the sensitivity of human gastric cancer (SGC7901) cells to chemotherapeutic drugs [36], and Kv1.1 specific blocker reduced gefitinib-resistant H460 cell viability [37]. Furthermore, inhibition of intermediate conductance calcium-activated potassium (KCa3.1) channels in a stem-like subpopulation from primary GBM cells induced reducing motility of stem-like subpopulation [38]. Kv channels are also involved in cell differentiation [39]. In particular, the expression of Kv1.3 was altered in poorly differentiated breast cancer [40], while Kv1.1, Kv1.2, Kv1.3, Kv1.4, Kv4.2, Kv4.3 and Kv9.3 decreased, as undifferentiated human mesenchymal stem cells (MSCs) differentiated into adipocytes [41]. In addition, the expression of neural Kv7 genes was increased during murine myoblast cell differentiation [42]. Likewise, our results also suggested that SP cells which were less sensitive to gefitinib have different mRNA expression patterns of Kv channel subtypes compared to Non-SP cells.

Over the last several years, in order to overcome the resistance of TKIs, many molecular targets have been investigated in cancer cells [43–45]. Kv channels as targets for suppression of cancer cell growth have been proposed [46–48]. Moreover, the inhibitory effect of combination therapies with EGFR TKIs and potassium channel blockers on the viability of cancer cells has been measured [37, 49]. However, the function of Kv channel blockers and a Kv7 opener on gefitinib-resistant SP cells has not been reported. According to our results, the combination treatment of gefitinib and Kv channel blockers or the Kv7 opener further attenuated the resistance of SP cells to gefitinib compared to single treatments. Therefore, our results demonstrate the synergic effect of gefitinib and Kv channel blockers (TEA and 4-AP) or the Kv7 opener (flupirtine) on the viability of gefitinib-resistant SP cells.

In addition, the signaling pathway related to reducing the resistance of SP cells to gefitinib with combination treatment of gefitinib and Kv channel blockers or the Kv7 opener has not been characterized. Recently, several reports have demonstrated that K^+^ channels are regulated by EGFR [50, 51]. Moreover, K^+^ currents have been induced by the Ras-Raf cascade [52]. Our study also suggests that the blockade or opening of Kv channels is associated with the EGFR-Ras-Raf signaling pathway.

## Conclusion

The SP cells in the NCI-H460 cell line have different Kv channel expressions compared to Non-SP cells, and the resistance of SP cells to gefitinib, with a highly expressed ABC transporter and a stemness gene, was attenuated through combination treatment with gefitinib and Kv channel blockers (TEA and 4-AP) or Kv7 opener (flupirtine). Therefore, the Kv channels of SP cells are useful targets for overcoming gefitinib resistance in lung cancer patients.

## Additional file

Additional file 1: Effect of gefitinb 1 μM on sorted SP cells and Non-SP cell viability. Sorted SP and Non-SP cells were seeded at 1 × 10^3^ cells in 96 well culture plates. After 24 h, cells were treated with gefitinib 1 μM. Cell viability was measured after 72 h. Results are mean ± SE of triplicate experiments. (PPTX 100 kb)

## Abbreviations

4-AP: 4-aminopyridine
ABC: ATP-binding cassette
BCNU: 1,3-Bis(2-chloroethyl)-1-nitrosourea
CLIC1: Chloride intracellular channel 1
CSCs: Cancer stem cells
EGFR: Epidermal growth factor receptor
GBM: Glioblastoma multiforme
MTT: 3-(4,5-dimethylthiazol-2-yl)-2,5-diphenyltetrazolium bromide
NSCLC: Non-small cell lung cancer
SP: Side-population
TEA: Tetraethylammonium
TKIs: Tyrosine kinase inhibitors
TRPM7: Transient receptor potential cation channel, subfamily M, member 7
