# Supplementary figures and images for: Regulation of voltage-gated potassium channels attenuates resistance of side-population cells to gefitinib in the human lung cancer cell line NCI-H460
Source: BMC Pharmacol Toxicol. 2017 Feb 21;18:14. doi: 10.1186/s40360-017-0118-9 (PMC5319158; doi:10.1186/s40360-017-0118-9)

## Slide 1
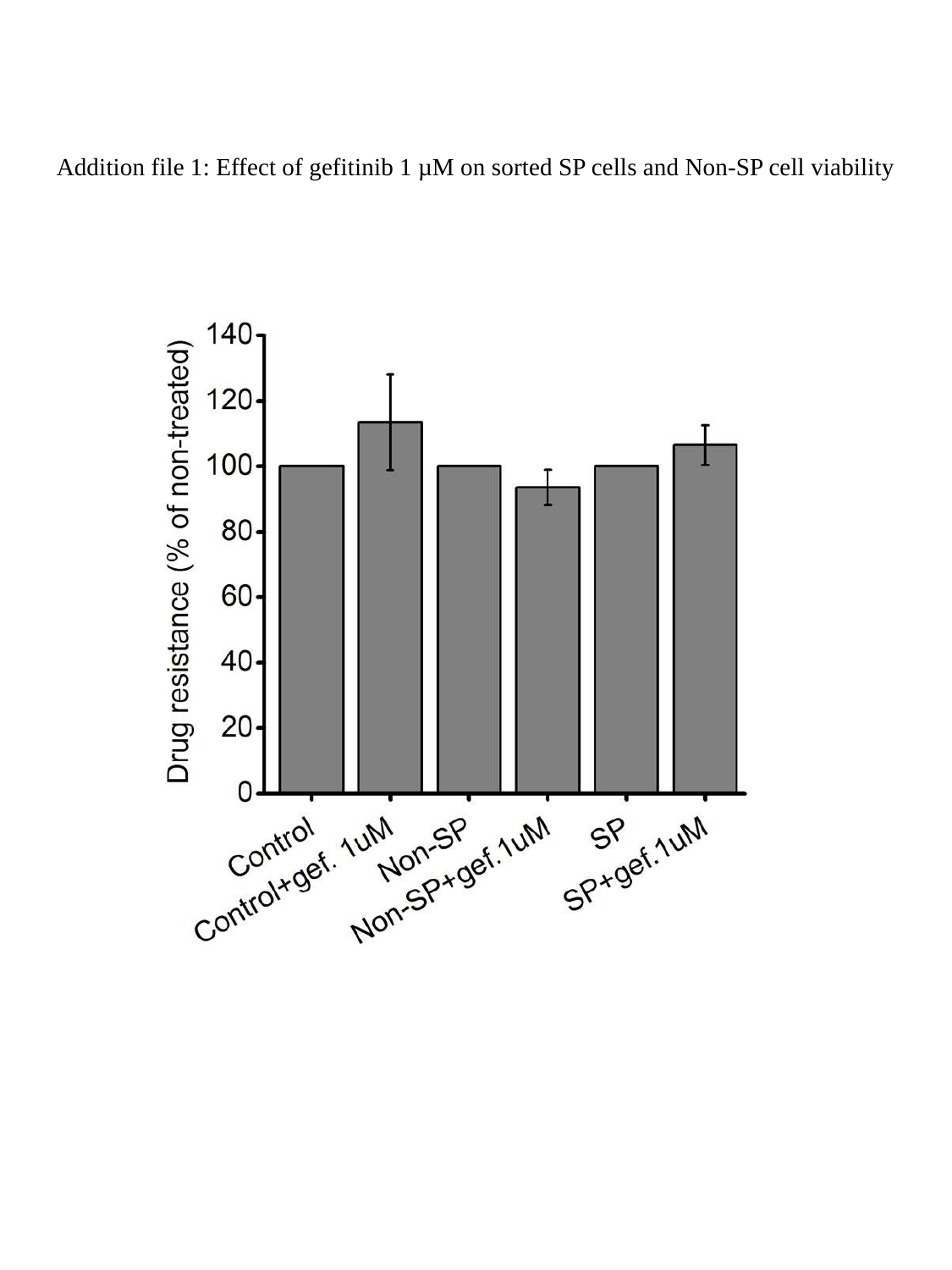

Addition file 1: Effect of gefitinib 1 µM on sorted SP cells and Non-SP cell viability

Supplement: Additional file 1: — Effect of gefitinb 1 μM on sorted SP cells and Non-SP cell viability. Sorted SP and Non-SP cells were seeded at 1 × 10^3^ cells in 96 well culture plates. After 24 h, cells were treated with gefitinib 1 μM. Cell viability was measured after 72 h. Results are mean ± SE of triplicate experiments. (PPTX 100 kb) [file 40360_2017_118_MOESM1_ESM.pptx]
